# Histone Deubiquitinase OTU1 Epigenetically Regulates *DA1* and *DA2*, Which Control *Arabidopsis* Seed and Organ Size

**DOI:** 10.1016/j.isci.2020.100948

**Published:** 2020-02-28

**Authors:** Ido Keren, Benoît Lacroix, Abraham Kohrman, Vitaly Citovsky

**Affiliations:** 1 Department of Biochemistry and Cell Biology, State University of New York, Stony Brook, NY 11794-5215, USA; 2 Graduate Program in Genetics, State University of New York, Stony Brook, NY 11794-5222, USA

**Keywords:** Plant Biology, Plant Genetics, Plant Development

## Abstract

Seeds are central to plant life cycle and to human nutrition, functioning as the major supplier of human population energy intake. To understand better the roles of enzymic writers and erasers of the epigenetic marks, in particular, histone ubiquitylation and the corresponding histone modifiers, involved in control of seed development, we identified the otubain-like cysteine protease OTU1 as a histone deubiquitinase involved in transcriptional repression of the *DA1* and *DA2* genes known to regulate seed and organ size in *Arabidopsis*. Loss-of-function mutants of *OTU1* accumulate H2B monoubiquitylation and such euchromatic marks as H3 trimethylation and hyperacetylation in the *DA1* and *DA2* chromatin. These data advance our knowledge about epigenetic regulation of the *DA1* and *DA2* genes by recognizing OTU1 as a member of a putative repressor complex that negatively regulates their transcription.

## Highlights

• Histone ubiquitylation regulates transcription of *DA1/DA2* that control seed/organ size
• OTU1 deubiquitinase is involved in deubiquitylation of the *DA1/DA2* chromatin
• OTU1 acts as an epigenetic transcriptional repressor of the *DA1/DA2* genes
• OTU1 is nucleocytoplasmic, indicating involvement in nuclear and cytoplasmic processes

## Graphical Abstract

**Figure:**
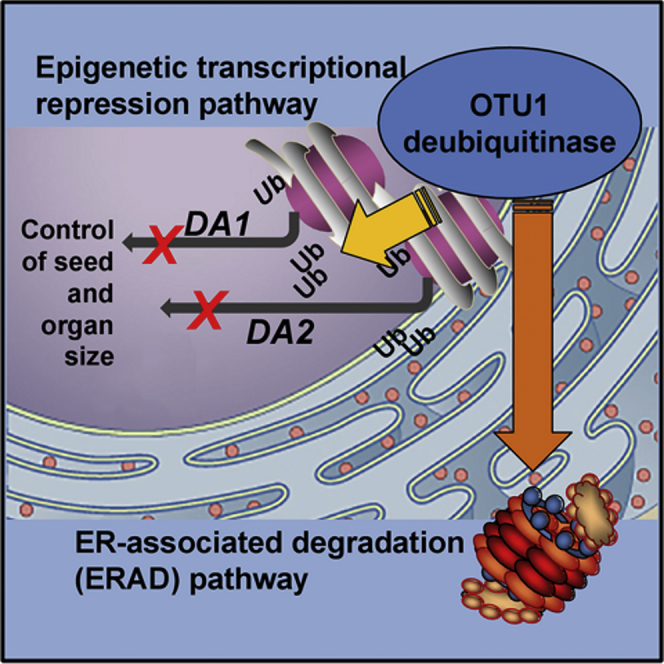

## Introduction

Monoubiquitylation of histone 2 molecules has been implicated in epigenetic regulation of many important aspects of plant life cycle. For example, H2B monoubiquitylation affects plant growth, seed dormancy, root and leaf growth, circadian clock, timing of flowering, and photomorphogenesis (Bourbousse et al., 2012, Fleury et al., 2007, Gu et al., 2009, Himanen et al., 2012, Keren and Citovsky, 2016, Keren and Citovsky, 2017, Liu et al., 2007). H2 monoubiquitylation, in turn, affects methylation and acetylation states of histones 3 and 4, ultimately resulting in transcriptional repression or activation of the corresponding genes (March and Farrona, 2018, Weake and Workman, 2008). This epigenetic pathway is regulated by histone deubiquitinase enzymes that erase the monoubiquityl marks from the histone molecules. The genome of the model plant *Arabidopsis* encodes five families of deubiquitinases, i.e., ubiquitin-specific proteases/processing proteases (USPs/UBPs), ubiquitin carboxy-terminal (UCH) proteases, Machado-Joseph disease protein domain proteases (MJD), JAB1/MPNC/MOV34 (JAMMs) proteases, and otubain-like cysteine proteases (OTU), that include approximately 60 members (Isono and Nagel, 2014, Komander et al., 2009, March and Farrona, 2018). Among these, only four enzymes, UBP26, UBP12, and UBP22, belonging to the USP/UBP family (Derkacheva et al., 2016, Feng and Shen, 2014, Isono and Nagel, 2014, Nassrallah et al., 2018) and only one enzyme, OTLD1, belonging to the OTU family (Keren and Citovsky, 2016, Keren and Citovsky, 2017, Krichevsky et al., 2011), have been demonstrated to use histones as substrate. We continued to study the OTU family, focusing on the OTU1 protein with no known phenotypic effects and functional roles. Using reverse genetics, we showed that OTU1 is a nucleocytoplasmic protein that affects the size of seeds and leaves and is involved in chromatin deubiquitylation and transcriptional repression of the *DA1* and *DA2* genes known to regulate seed and organ size in *Arabidopsis* (Du et al., 2014, Li and Li, 2014, Li and Li, 2016, Xia et al., 2013).

## Results

### OTU1 Is a Nucleocytoplasmic Protein

**Figure 1:**
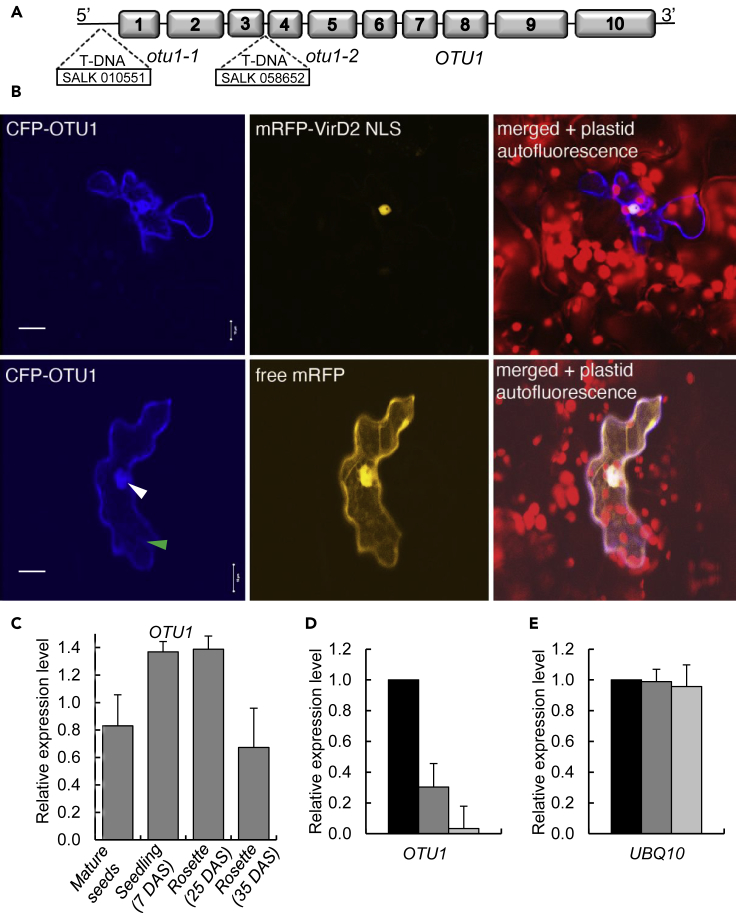
Loss-of-Function Alleles of OTU1 and Nucleocytoplasmic Localization of the OTU1 Protein (A) Schematic structure of the *OTU1* gene with the locations of the mutagenic transfer DNA insertions in the *otu1-1* and *otu1-2* mutants. Exons are indicated by sequentially numbered boxes. (B) Subcellular localization of OTU1 in *Arabidopsis* leaf epidermis. CFP signal is in blue, mRFP signal is in orange, and overlapping CFP/mRFP signals are in pink. Chloroplast autofluorescence is in red. Green arrowhead points to a cytoplasmic transvacuolar strand, and white arrowhead points to the cell nucleus. All images are single confocal sections. All images are representative of multiple independent experiments (N = 20 images from five plants). Scale bars, 10 μm. (C) Expression levels of the *OTU1* gene in seeds and aerial tissues of the wild-type *Arabidopsis* plants at different ages. DAS, days after seed stratification. Error bars represent SD; N = 2 independent biological replicates. (D) Reduced expression of the *OTU1* gene in the *otu1-1* and *otu1-2* plants. p = 0.05 for statistical significance of differences between the mutant and wild-type plants. (E) Expression of the reference gene *UBQ10* for the analysis shown in (D). Differences between all tested plants are not statistically significant (p > 0.05). Relative expression of *OTU1* in wild-type (black bars), *otu1-1* (dark gray bars), and *otu1-2* plants (light gray bars) was analyzed by RT-qPCR. The expression level in the wild-type plants is set to 1.0; error bars represent SD; N = 3 independent biological replicates.

OTU1 is an otubain-like histone deubiquitinase encoded by the *Arabidopsis At1g28120* gene (Isono and Nagel, 2014) (Figure 1A). OTU1 belongs to a 13-member family of *Arabidopsis* OTU deubiquitinases (Figure S1), most of which remain uncharacterized (Isono and Nagel, 2014, Komander et al., 2009). To determine its subcellular localization in plant cells, OTU1 was tagged with CFP and transiently expressed, following biolistic delivery of its encoding DNA construct, in the *Arabidopsis* leaf epidermis together with free monomeric red fluorescent protein (mRFP) reporter that partitions between the cell cytoplasm and the nucleus, conveniently visualizing and identifying both these cellular compartments. As positive control for nuclear localization, CFP-OTU1 was coexpressed with mRFP fused to a bipartite-type nuclear localization signal (NLS) derived from the *Agrobacterium* VirD2 protein (Howard et al., 1992). Figure 1B shows that, similarly to free mRFP, CFP-OTU1 accumulated in the cytoplasm—displaying transvacuolar strands (green arrowhead) and variations in cytosol thickness at the cell cortex (Cutler et al., 2000, Tian et al., 2004)—and in the nucleus (white arrowhead), colocalizing with mRFP-VirD2 NLS. Indeed, the combined images of CFP (blue color) and mRFP fluorescence (orange color) showed overlapping signal (pink color) within both the cell cytoplasm and the cell nucleus (Figure 1B). We cannot rule out that at least some of the cytoplasmic signal of CFP-OTU1 derives from degradation of this fusion protein; however, usually, degradation of GFP-tagged proteins results in the loss of fluorescence, representing the rationale for degradation assays that utilize GFP fusions of the proteins of interest (Gray et al., 2001, Tzfira et al., 2004, Wang et al., 2017). Furthermore, analysis of OTU1 using the Subcellular Localization Database for Arabidopsis proteins (SUBA) (Hooper et al., 2017) also suggested, through prediction and experimental data, nucleocytoplasmic localization with 65%/35% probability, respectively (http://suba.live/suba-app/factsheet.html?id=AT1G28120).

In addition to the subcellular localization of OTU1, we examined the expression pattern of the *OTU1* in the wild-type *Arabidopsis* plants using quantitative RT-PCR (RT-qPCR) analysis. We focused on seeds and aerial tissues of the wild-type plants of different ages, which corresponded to the organs most affected by the loss of function of *OTU1* (see below). This analysis showed that *OTU1* was expressed at higher levels in seedlings and younger rosette leaves and at lower levels in mature seeds and older rosettes (Figure 1C).

### *OTU1* Loss of Function Affects the Size of Seeds, Leaf Rosettes, and Stems

We examined two available *Arabidopsis* transfer DNA insertion mutants, *otu1-1* and *otu1-2*, homozygous for transfer DNA insertion into the *OTU1* gene. In *otu1-1*, the mutagenic insert is located in the 5′ UTR, and in *otu1-2*, the mutagenic insert is located between the third and the fourth exons (Figure 1A). The RT-qPCR analysis showed that the *otu1-1* plants expressed *OTU1* at significantly lower levels relative to the wild-type plants (Figure 1D), whereas in the *otu1-2* plants, the OTU1 transcripts were barely detected; in the internal control, the *UBQ10* reference gene displayed similar expression levels in all plant lines (Figure 1E).

**Figure 2:**
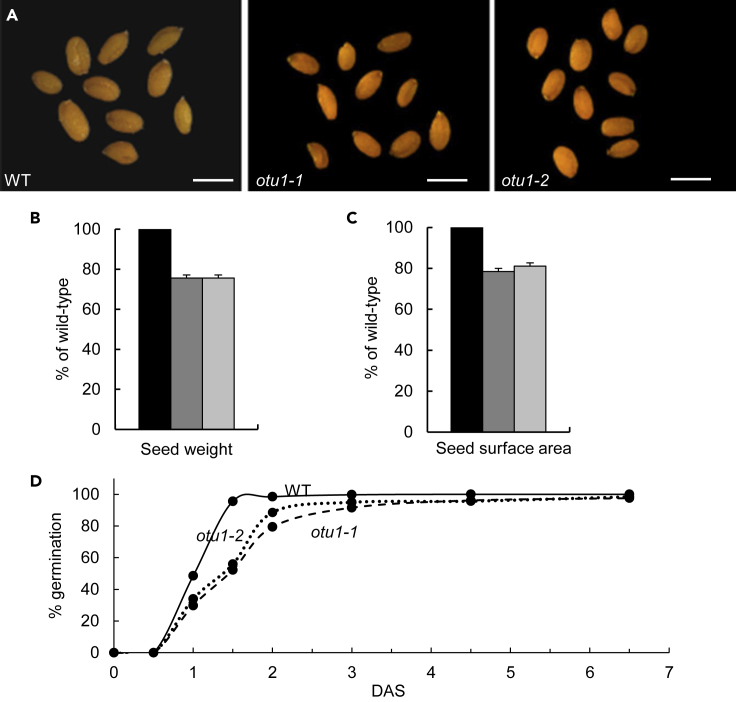
Reduced Seed Size in *otu1-1* and *otu1-2* Plants (A) Seeds of the indicated plant lines. Scale bars, 0.5 mm. (B) Seed weight (N = 500 seeds from each line). (C) Seed surface area (N = 100 seeds from each line). WT (wild-type) plants, black bars; *otu1-1*, dark gray bars; *otu1-2*, light gray bars. Error bars represent SD. p = 0.05 for statistical significance of differences in seed parameters between the mutant and wild-type plants; differences between *otu1-1* and *otu1-2* plants are not statistically significant (p > 0.05). (D) Time course for seed germination (N = 500 seeds from each line). The solid, dashed, and dotted lines represent WT, *otu1-1*, and *otu1-2* lines, respectively. DAS, days after seed stratification. Differences in germination at 1.5 DAS, corresponding to the linear part of the *otu1-1* and *otu1-2* germination kinetics corresponded to p = 0.05.

**Figure 3:**
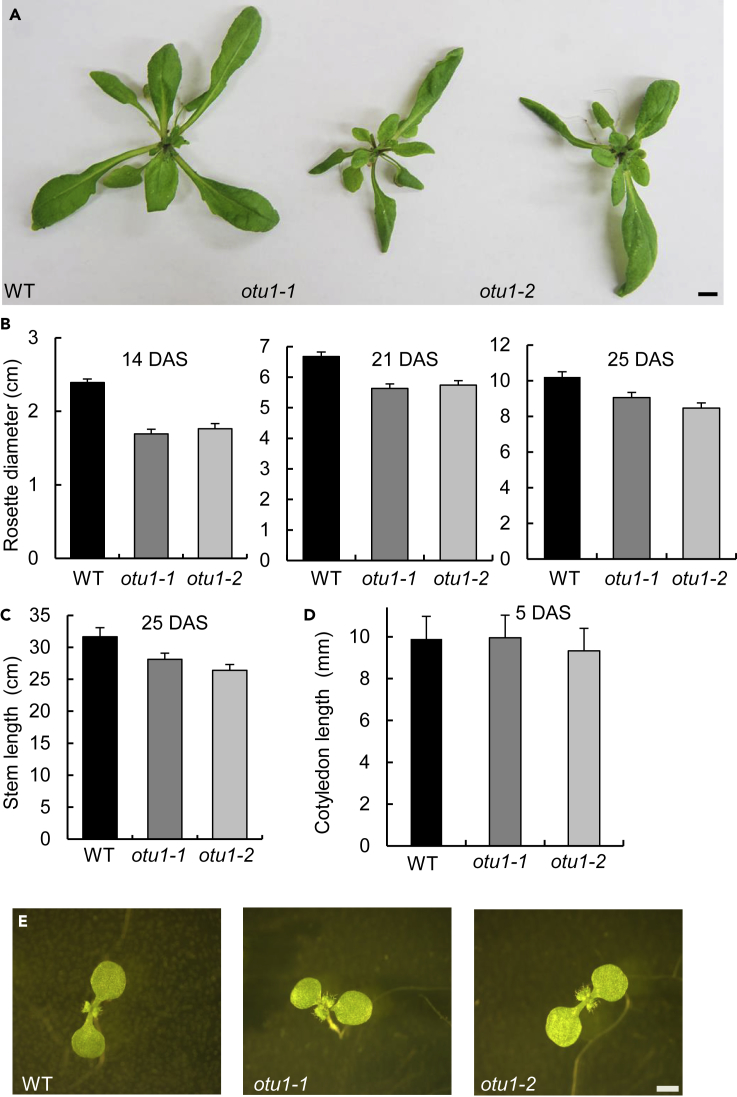
Reduced Leaf Rosette and Stem Size in *otu1-1* and *otu1-2* Plants (A) Representative rosettes at 21 DAS. Scale bar = 10 mm. (B) Rosette diameter at the indicated DAS (N = 50 plants). (C) Stem length (N = 35 plants). p = 0.05 for statistical significance of differences in seed parameters between the mutant and wild-type plants; differences between *otu1-1* and *otu1-2* plants are not statistically significant (p > 0.05). (D) Cotyledon length (N = 10 plants). WT (wild-type) plants, black bars; *otu1-1*, dark gray bars; *otu1-2*, light gray bars. Error bars represent SD. Differences between the mutant and wild-type plants are not statistically significant (p > 0.05). (E) Cotyledons at 7 DAS. Scale bar, 1 mm. DAS, days after seed stratification.

Next, we assessed the overall phenotypic effects of the *otu1-1* and *otu1-2* loss-of-function mutations. Both mutant lines exhibited two readily detectable alterations in their morphology: reduced seed size (Figure 2A) and reduced leaf rosette diameter and stem length (Figures 3A–3C). Seeds produced by the *otu1-1* and *otu1-2* plants were lighter and smaller than the wild-type seeds (Figures 2B and 2C). Time course studies indicated slower rate of germination of seeds from both *otu1-1* and *otu1-2* plants when compared with the wild-type plants; for example, the wild-type seeds reached 96% germination already after 36 h, at which time only about 52% and 56% of the *otu1-1* and *otu1-2* seeds, respectively, germinated (Figure 2D). However, as time progressed, the *otu1-1* and *otu1-2* seeds continued to germinate, catching up with the wild-type seeds after 6.5 days when seeds from all three plant lines reached ca. 98% germination (Figure 2D).

**Figure 4:**
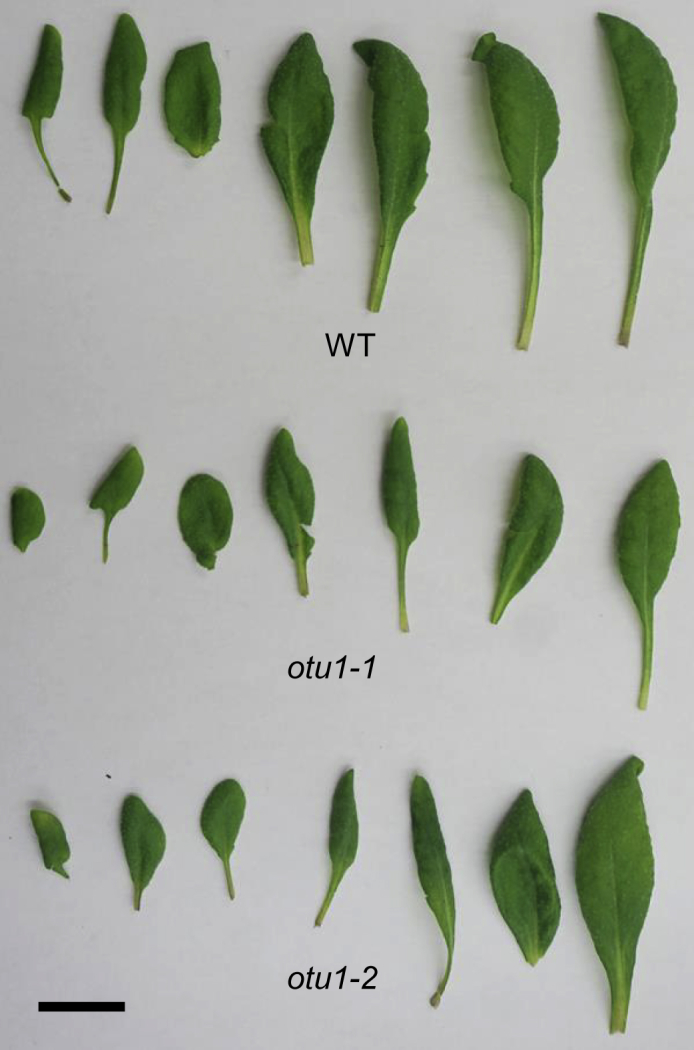
Leaf Size Distribution in Rosettes of *otu1-1* and *otu1-2* Plants Each of the seven sequential leaves from a rosette of the indicated plant lines was removed and arranged right to left sequentially for size comparison. Images are representative of multiple independent experiments (N = 10 images from three plants of each line). Scale bar, 5.0 mm.

The *otu1-1* and *otu1-2* plants also developed smaller leaf rosettes (Figure 3A). Quantification of the rosette diameter showed that leaf rosettes of both mutants were smaller than those of the wild-type plants, and that this difference gradually diminished with plant age (Figure 3B). As expected, the size of the individual leaves in the rosettes from both the *otu1-1* and *otu1-2* mutant plants was smaller than the size of the corresponding leaves from the wild-type plants (Figure 4). Also, we observed modest reduction in the length of plant stems between the wild-type and the *otu1-1* and *otu1-2* mutants (Figure 3C). Similar to the seed size observations, we did not detect differences in the rosette diameter and stem length between the *otu1-1* and *otu1-2* plants (Figures 3B and 3C). Also, no differences in size of cotyledons were observed between both mutants and the wild-type plants (Figures 3D and 3E).

**Figure 5:**
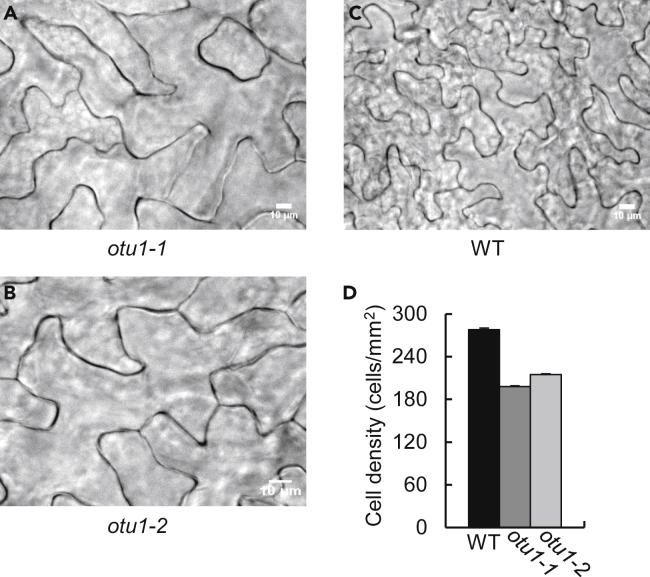
Increased Expansion and Reduced Proliferation of Leaf Epidermal Cells in *otu1-1* and *otu1-2* Plants (A–C) DIC images of epidermal cells in fully-expanded fifth leaves of the *otu1-1* (A), *otu1-2* (B), and wild-type (WT) plants (C), respectively, at 21 DAS. Cells of both mutants are clearly larger and fewer per microscope field than the cells of the wild-type plant. DAS, days after seed stratification. Scale bars, 10 μm. (D) Cell density at leaf epidermal midrib. WT, black bars; *otu1-1*, dark gray bars; *otu1-2*, light gray bars. Error bars represent SD, N = 9 images from three independent plants per line.

The reduced organ size of the *otu1-1* and *otu1-2* plants could result from a decrease in cell number, cell size, or both. To assess such possible contributions of cell proliferation and/or expansion, we examined the size and surface density of adaxial epidermal cells of fully expanded fifth rosette leaves known to represent faithfully the characteristic features of rosette leaf development in *Arabidopsis* (Tsuge et al., 1996). Count of cells in the blade midrib sections of the leaf revealed that their surface density in both *otu1-1* and *otu1-2* lines was lower (Figure 5), by ca. 22%–30% of the wild-type leaves (Figure 5D). The size distribution of epidermal cells in these areas was slightly enlarged when compared with the wild-type plants (Figures 5A–5C), i.e., averaging 5,000 μm^2^ and 4,600 μm^2^ in the *otu1-1* and *otu1-2* plants versus 3,600 μm^2^ in the wild-type plants; we focused on cell size distribution because it is known to be highly reproducible in *Arabidopsis* leaves, whereas the size of individual cells can vary (Kawade and Tsukaya, 2017). Thus, the loss of function of *OTU1* likely reduces cell proliferation but promotes cell expansion.

### OTU1 Is Involved in Transcriptional Repression of the *DA1* and *DA2* Genes

**Figure 6:**
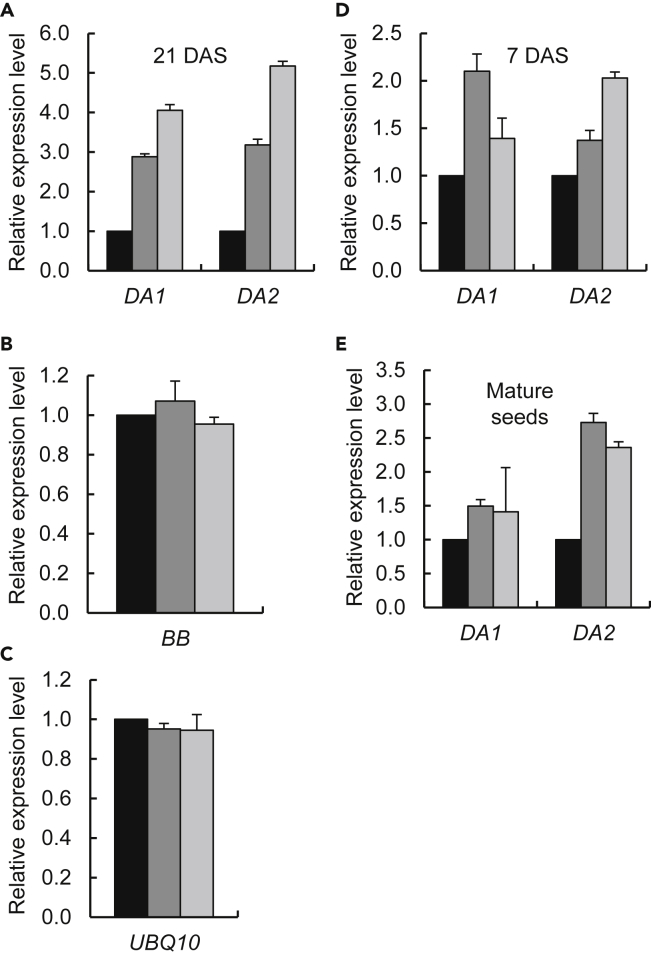
Transcriptional Activation of *DA1* and *DA2* Genes in *otu1-1* and *otu1-2* Plants (A) Elevated expression of the *DA1* and *DA2* genes in the *otu1-1* and *otu1-2* rosette-leaves at 25 DAS. p = 0.05 for statistical significance of differences between the mutant and wild-type plants. (B) Expression of the gene *BB* for the analysis shown in (A). (C) Expression of the reference gene *UBQ10* for the analysis shown in (A). Differences between all tested plants are not statistically significant (p > 0.05). (D) Elevated expression of the *DA1* and *DA2* genes in the *otu1-1* and *otu1-2* seedlings at 7 DAS. DAS, days after seed stratification. (E) Elevated expression of the *DA1* and *DA2* genes in the *otu1-1* and *otu1-2* mature seeds. p = 0.05 for statistical significance of differences between the mutant and wild-type plants. Relative expression of *OTU1* in wild-type (black bars), *otu1-1* (dark gray bars), and *otu1-2* tissues (light gray bars) was analyzed by RT-qPCR. The expression level in the wild-type plants is set to 1.0; error bars represent SD; N = 3 independent biological replicates.

The major phenotypic hallmarks of the *otu1-1* and *otu1-2* plants, i.e., reduced size of seeds and of several aerial organs such as leaves and stems, inform about the possible identity of the target genes of OTU1 and facilitate their rational prediction by the inductive approach. Specifically, we focused on the three main molecular pathways, the ubiquitin-proteasome-based pathway, the transcription factor-based pathway, and the IKU pathway, that regulate seed size through three distinct processes, cell proliferation, cell expansion, and precocious endosperm cellularization, respectively (Li and Li, 2016). We then selected six genes that represent some of the major participants of each of these pathways (Table S1) and tested whether any of them exhibited altered expression in the mutant lines. To this end, transcript levels of each of these genes were analyzed by RT-qPCR in the rosette leaves of the *otu1-1* and *otu1-2* plants and compared with the wild-type plants (Figure 6). Most of the tested genes showed no significant changes in their expression levels in any of the plant lines; this group of genes is exemplified by *BIG BROTHER* (*BB*), a negative regulator of seed size (Vanhaeren et al., 2017), the transcripts of which accumulated to comparable amounts in the *otu1-1*, *otu1-2*, and wild-type plants (Figure 6B). However, two genes, *DA1* and *DA2*, displayed substantial increase in expression in both loss-of-function lines (Figure 6A). Specifically, *DA1* transcript amounts in the rosette leaves were elevated ca. 3- to 4-fold in *otu1-1* and *otu1-2*, respectively, whereas the levels of the *DA2* transcript increased ca. 3- to 5-fold in the same plants (Figure 6A). The expression of the internal reference gene *UBQ10* was not altered in any of the plant lines (Figure 6C). We then examined *DA1* and *DA2* expression in the seedlings and in mature seeds of both mutant lines. We observed ca. 1.5- to 2-fold enhanced levels of *DA1* and *DA2* transcripts in *otu1-1* and *otu1-2* seedlings, respectively, when compared with the wild-type seedlings (Figure 6D). In mature *otu1-1* and *otu1-2* seeds, expression of both *DA1* and *DA2* genes was elevated by ca. 1.5- and 2.5-3 fold, respectively (Figure 6E). These data suggest that OTU1 negatively regulates expression of *DA1* and *DA2* and that its loss of function results in transcriptional activation of these target genes.

DA1, a ubiquitin-binding protein, and DA2, a RING-type E3 ubiquitin ligase, are known to interact with each other and negatively regulate the seed and organ size in *Arabidopsis* (Du et al., 2014, Li and Li, 2014, Li and Li, 2016, Xia et al., 2013). Thus, elevated expression of *DA1* and *DA2* in the *otu1-1* and *otu1-2* mutants is expected to decrease the seed and organ size, consistent with the phenotypes observed in these plants (see Figures 2 and 3).

### OTU1 Is Involved in Deubiquitylation of the *DA1* and *DA2* Chromatin

**Figure 7:**
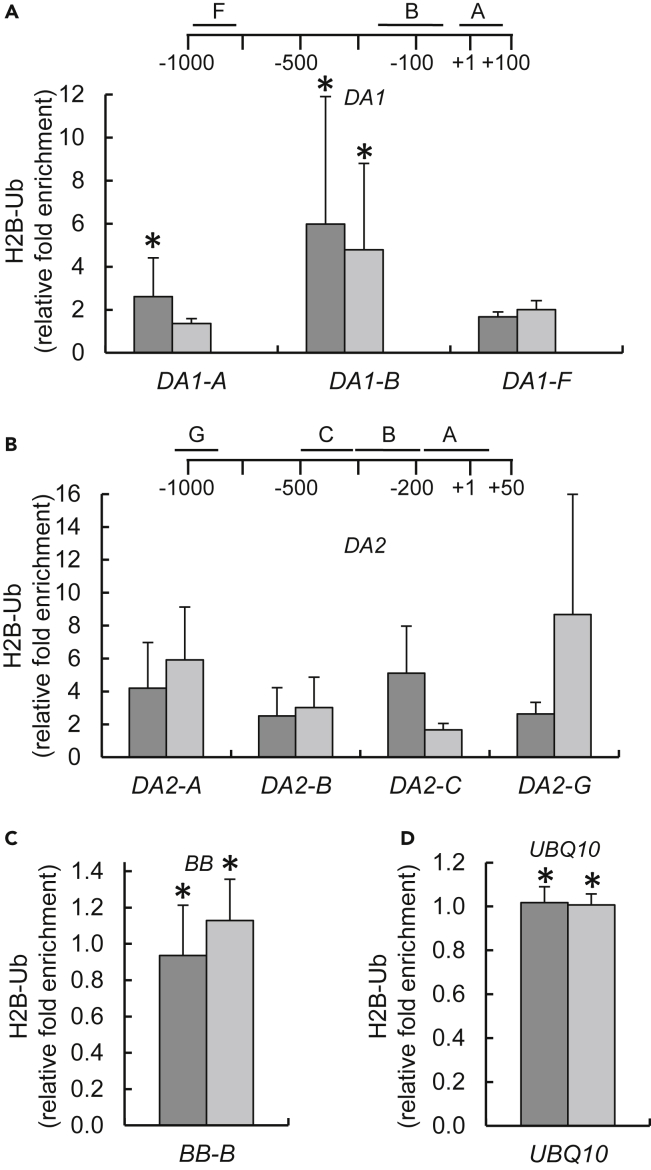
H2B hyperubiquitylation in *DA1* and *DA2* Gene Chromatin in *otu1-1* and *otu1-2* Plants (A–D) qChIP analyses of relative levels of H2B monoubiquitylation in the mutant relative to the wild-type plants are shown for (A) *DA1*, (B) *DA2*, (C) *BB*, and (D) *UBQ10*. Locations of sequences relative to the translation initiation site (ATG) used for qChIP analyses are indicated for each gene and detailed in Table S1. *otu1-1*, dark gray bars; *otu1-2*, light gray bars. Error bars represent SD; N = 3 independent biological replicates; p = 0.05 for statistical significance of differences between the mutant and wild-type plants, except where indicated by asterisks, which denote differences that are not statistically significant (p > 0.05) as determined by Wilcoxon signed-rank tests. Differences between the *otu1-1* and *otu1-2* plants were statistically insignificant (p = 0.2–1.0).

Increased H2B monoubiquitylation often induces gene expression (Batta et al., 2011, Shukla and Bhaumik, 2007, Tanny et al., 2007, Weake and Workman, 2008). It makes biological sense, therefore, that OTU1 acts to deubiquitylate H2B in the target genes' chromatin; in this scenario, H2B monoubiquitylation in the *DA1* and *DA2* chromatin should increase in the *OTU1* loss-of-function mutants. We examined this notion using quantitative chromatin immunoprecipitation (qChIP). Our qChIP analysis showed substantial levels of hyperubiquitylation of H2B in the *DA1* and *DA2* chromatin (Figure 7). Specifically, we detected two regions in the *DA1* chromatin and four regions in the *DA2* chromatin of *otu1-1* and/or *otu1-2*, which were located upstream of and flanked the translation initiation codon, with monoubiquitylation amounts ranging between ca. 1.5- and 8-fold higher than the wild-type *DA1* and *DA2* chromatin (Figures 7A and 7B). Consistent with the effect of the *OTU1* loss-of-function mutations on the *DA1* and *DA2* transcription (see Figure 6), their effect on *DA2* monoubiquitylation was more pronounced than that on *DA1* monoubiquitylation. Confirming the specificity of these observations, no significant changes in the degree of H2B monoubiquitylation were detected in the chromatin of *BB*, the expression of which was not altered by loss of function of *OTU1* (see Figure 6B), or in the chromatin of the *UBQ10* reference gene (Figures 7C and 7D). Thus, OTU1 most likely specifically deubiquitylates chromatin of its target genes, rather than acting as a general modifier of chromatin.

### OTU1 Loss of Function Promotes Increase in Euchromatic Histone Methylation and Acetylation Marks

**Figure 8:**
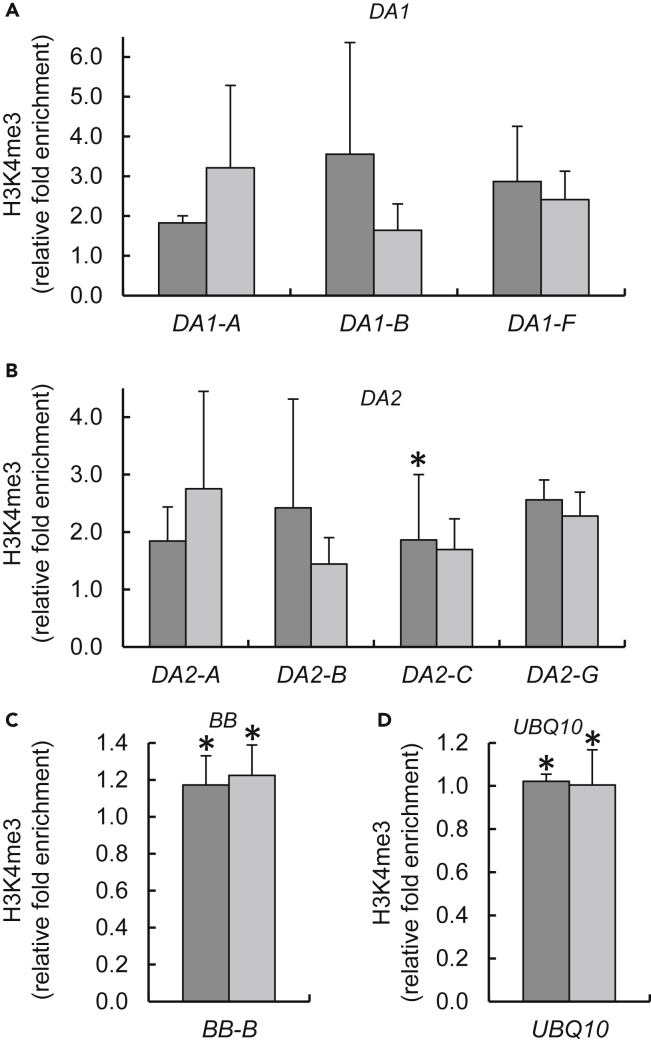
Increase in Trimethylation of H3K4 in *DA1* and *DA2* Gene Chromatin in *otu1-1* and *otu1-2* Plants (A–D) qChIP analyses of relative levels of H3K4me3 in the mutant relative to the wild-type plants are shown for (A) *DA1*, (B) *DA2*, (C) *BB*, and (D) *UBQ10*. Locations of sequences relative to the translation initiation site (ATG) used for qChIP analyses are diagrammed in Figure 7 and detailed in Table S1. *otu1-1*, dark gray bars; *otu1-2*, light gray bars. Error bars represent SD; N = 3 independent biological replicates; p = 0.05 for statistical significance of differences between the mutant and wild-type plants, except where indicated by asterisks, which denote differences that are not statistically significant (p > 0.05) as determined by Wilcoxon signed-rank tests. Differences between the *otu1-1* and *otu1-2* plants were statistically insignificant (p = 0.4–1.0).

H2B deubiquitylation has been shown to facilitate removal of euchromatic histone modification marks (Cao et al., 2008, Gu et al., 2009, Sridhar et al., 2007), of which some of the major ones are H3K4me3 and H3Ac. Thus, increase in H2B monoubiquitylation is expected to elicit a reverse effect on these marks. We used qChIP to analyze, in the *otu1-1* and *otu1-2* plants, the chromatin of the *DA1* and *DA2* genes for possible changes in their H3K4me3 and H3Ac contents. The chromatin of the *DA1* and *DA2* genes contained higher levels of H3K4me3 (Figures 8A and 8B). Specifically, the H3K4me3 content of the *DA1* chromatin of both the *otu1-1* and *otu1-2* lines was ca. 2- to 3.5-fold higher, depending on the tested chromatin region, than that of the wild-type plants (Figure 8A), in the same chromatin regions found to be hyperubiquitylated (see Figure 7A). Similarly, in the same plants, the trimethylation of H3K4 of the *DA2* chromatin was elevated by ca. 2- to 2.5-fold (Figure 8B, regions A and G). As expected, changes in the extent of H3K4 trimethylation of the chromatin of the *BB* gene and of the internal reference *UBQ1*0 gene were insignificant (Figures 8C and 8D).

**Figure 9:**
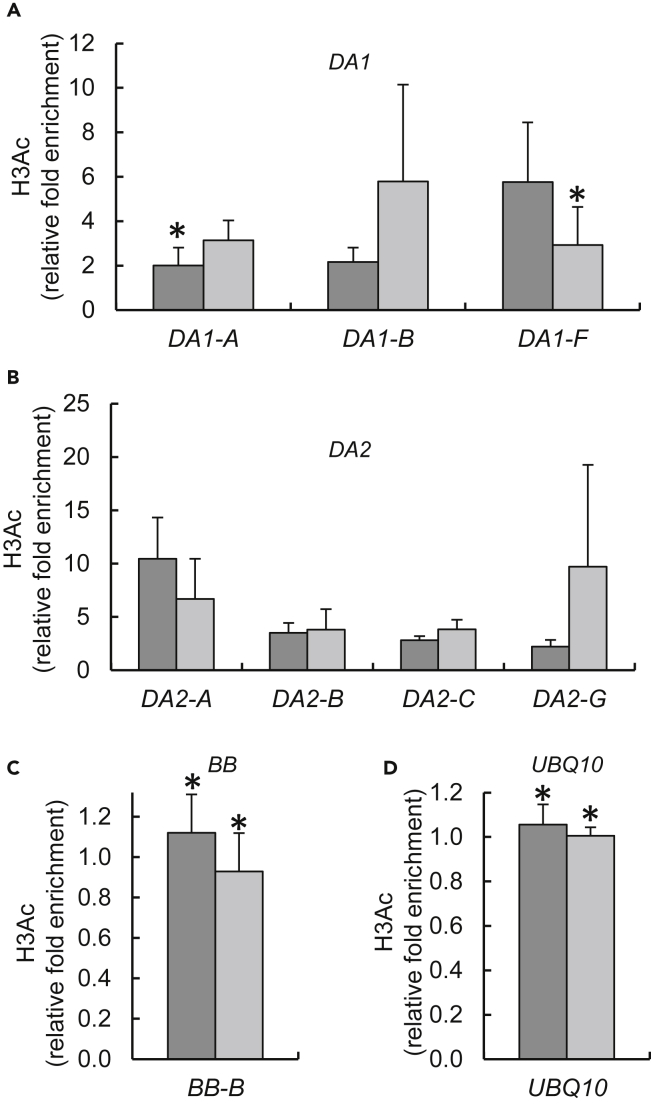
Hyperacetylation of H3 in *DA1* and *DA2* Gene Chromatin in *otu1-1* and *otu1-2* Plants (A–D) qChIP analyses of relative levels of H3 acetylation in the mutant relative to the wild-type plants are shown for (A) *DA1*, (B) *DA2*, (C) *BB*, and (D) *UBQ10*. Locations of sequences relative to the translation initiation site (ATG) used for qChIP analyses are diagrammed in Figure 7 and in Table S1. *otu1-1*, dark gray bars; *otu1-2*, light gray bars. Error bars represent SD; N = 3 independent biological replicates; p = 0.05 for statistical significance of differences between the mutant and wild-type plants, except where indicated by asterisks, which denote differences that are not statistically significant (p > 0.05) as determined by Wilcoxon signed-rank tests. Differences between the *otu1-1* and *otu1-2* plants were statistically insignificant (p = 0.4–0.7).

We also observed histone hyperacetylation in the *DA1* and *DA2* chromatin in the *otu1-1* and *otu1-2* mutants (Figures 9A and 9B). In several regions of the *DA1* chromatin tested, the H3 acetylation increased by ca. 2- to 6-fold (Figure 9A). In the *DA2* chromatin, the acetylation levels increased by ca. 2- to 10-fold (Figure 9B). These changes were specific because, in negative control experiments, no significant changes in H3 acetylation were observed in the chromatin of *BB* (Figure 9C), the expression of which was not affected in the *otu1-1* and *otu1-2* lines (see Figure 6B) or in the chromatin of the *UBQ10* reference gene (Figure 9D). Collectively, our data suggest that OTU1 may act as transcriptional repressor of the DA1 and D2 genes, known repressors of the seed and organ size in *Arabidopsis*.

## Discussion

Seeds are central to plant reproduction and human nutrition, accounting for approximately 70% of energy intake of human population (Sreenivasulu and Wobus, 2013). Thus, seed development has been a subject of numerous studies, for many decades, uncovering multiple and diverse pathways for its control. For example, the ubiquitin/proteasome system (UPS) and G protein, mitogen-activated protein kinase, and brassinosteroid signaling pathways regulate seed size by affecting cell proliferation and expansion (Li and Li, 2016). Transcriptional control also plays an important role in seed development, involving different transcription factors and chromatin-modifying enzymes (Khan et al., 2014, Li and Li, 2016, Sun et al., 2010, Wang and Köhler, 2017). Yet, our knowledge of histone post-translational modifications and enzymic writers and erasers of epigenetic marks controlling seed development is largely lacking, so far limited to several polycomb (PcG) proteins and other histone methyltransferases and histone acetyltransferases (Li and Li, 2016, Sun et al., 2010). In particular, it remains unknown whether histone ubiquitylation and the corresponding histone modifiers have any role in controlling seed size. This knowledge gap contrasts our detailed understanding of ubiquitin-mediated control of seed size, which mainly focuses on UPS components (Li and Li, 2014). Here, we provide evidence for the involvement of H2B deubiquitylation and the specific histone deubiquitinase OTU1 in control of seed and organ size. OTU1 belongs to the *Arabidopsis* OTU family of deubiquitinases, which contains 13 proteins (Isono and Nagel, 2014, Komander et al., 2009). So far, only two members of this enzyme family have been functionally characterized, OTU5 shown to be involved in root responses to phosphate starvation (Suen and Schmidt, 2018, Suen et al., 2018, Yen et al., 2017) and OTLD1 shown to be involved in plant growth (Keren and Citovsky, 2016, Keren and Citovsky, 2017, Krichevsky et al., 2011). Furthermore, of these two enzymes, only OTLD1 has been demonstrated to function as a histone deubiquitinase and epigenetic regulator of a series of target genes involved in organ growth and development (Keren and Citovsky, 2016, Keren and Citovsky, 2017, Krichevsky et al., 2011). Our data suggest that OTU1 is also a histone deubiquitinase, the target genes of which include *DA1* and *DA2*, the major regulators of seed and organ size (Du et al., 2014, Li and Li, 2014, Li et al., 2008, Vanhaeren et al., 2017, Xia et al., 2013).

DA1 is a ubiquitin receptor that interacts with DA2, a RING-type E3 ubiquitin ligase, targeting specific substrates for degradation. Although most of these substrates remain unidentified, DA1 has been shown to recognize UBIQUITIN–SPECIFIC PROTEASE 15 (UBP15) and modulate its stability (Li and Li, 2014, Li and Li, 2016). Overexpression of *DA1* or *DA2* results in reduced seed and organ growth (Vanhaeren et al., 2017, Xia et al., 2013), and their loss-of-function mutants produce larger seeds (Xia et al., 2013). Thus, increased expression of *DA1* and *DA2* in the *otu1-1* and *otu1-2* mutants most likely underlies the reduced seed, rosette, and stem size observed in these plants. Interestingly, DA1 regulates the seed and organ size synergistically with another RING-type E3 ubiquitin ligase, BB (Li and Li, 2014, Li and Li, 2016, Li et al., 2008, Vanhaeren et al., 2017). However, whereas, consistent with their physical interaction, DA1 and DA2 may control seed size via the same pathway, genetic analyses suggested that DA2 and BB act in different pathways (Li and Li, 2016, Xia et al., 2013). The fact that the loss of *OTU1* function did not affect expression of *BB* in the *otu1-1* and *otu1-2* plants indicates that these two ubiquitin-mediated pathways are transcriptionally regulated by different chromatin modifiers. For *DA1* and *DA2*, their transcription is most likely regulated by OTU1 that acts as a transcriptional corepressor, deubiquitylating the *DA1* and *DA2* chromatin. In the absence of OTU1, the *DA1* and *DA2* chromatin accumulates H2B monoubiquitylation and such euchromatic marks as H3 trimethylation and hyperacetylation.

Although the molecular pathways by which DA1 and DA2 regulate the seed and organ size have been studied (Li and Li, 2014, Li and Li, 2016), regulation of expression of the *DA1* and *DA2* genes themselves has not been examined. Our data began filling this gap by identifying OTU1 as a member of a putative repressor complex that negatively regulates *DA1* and *DA2* transcription. Interestingly, OTU1 exhibits nucleocytoplasmic distribution in the cell. Obviously, nuclear localization of OTU1 is consistent with its biological function as histone deubiquitinase. On the other hand, the cytoplasmic location suggests an additional, non-nuclear, function for OTU1 in other cellular processes, potentially unrelated to chromatin remodeling and with non-histone substrates. Indeed, a recent study reported that OTU1 functions in the endoplasmic reticulum (ER)-associated degradation (ERAD) (Zang et al., 2020). Although this study did not examine the OTU1 subcellular localization directly, the ER-based function suggests that the cytoplasmic OTU1, at least in part, associates with the ER. Because our data detected OTU1 in the cell cytoplasm as mostly cytosolic, e.g., in the transvacuolar strands, the putative ER-associated population of OTU1 most likely is masked by its cytosolic pool. Taken together our data and the study by Zang et al. (2020) suggest a dual function for OTU1 in the plant cell: a histone deubiquitinase involved in transcriptional repression of its target genes and a protein deubiquitinase involved in processing of ERAD substrates. These findings underscore one apparent difference between the plant, animal, and yeast OTU-type deubiquitinases. At least two plant OTU families members, OTLD1 (Keren and Citovsky, 2016, Keren and Citovsky, 2017, Keren et al., 2019, Krichevsky et al., 2011) and OTU1, are involved in epigenetic regulation of transcription by histone deubiquitylation, with one of them, OTU1, also deubiquitylating other substrates and involved in a transcription-unrelated process of ERAD (Zang et al., 2020). In contrast, to our knowledge, animal and yeast OTU family members have not been shown to deubiquitylate histones, and they are involved in diverse cellular processes that do not include direct epigenetic transcriptional control, acting, for example, to stabilize their targets, such as the non-canonical nuclear factor-κB pathway component TRAF3 for human OTUD7B (Hu et al., 2013), by removal of ubiquitin residues thereby protecting them from proteasomal degradation, or by regulating the activity of their targets, such as the E3 ligase RNF168 and E2 ligase UBE2E1 for human OTUB1 (Nakada et al., 2010, Pasupala et al., 2018), in a proteasome-independent or even non-catalytic manner.

### Limitations of the Study

Our study demonstrates involvement of histone ubiquitylation chromatin marks and their erasure by histone deubiquitinase OTU1 in control of two genes, *DA1* and *DA2*, that are central to controlling seed and organ size in *Arabidopsis*. It remains to be investigated whether OTU1 itself is physically associated with the target chromatin, e.g., the promoter regions of *DA1* and *DA2*; furthermore, a global gene chromatin association study is required for exhaustive identification of *Arabidopsis* genes directly regulated by OTU1. Because OTU1 does not have DNA-binding domains, it presumably acts as a corepressor, requiring a DNA-binding transcription factor for specific recruitment to the target chromatin; identification of such putative transcription factor(s) also awaits further studies. Finally, OTU1 appears to participate in two different regulatory pathways that take place in different cellular locations: epigenetic regulation of gene expression in the nucleus and proteasomal degradation of misfolded proteins of the ER. It would be useful to define the cellular cues that determine which population of OTU1 molecules is targeted to the cell nucleus for histone deubiquitylation and which remains in the cell cytoplasm for participation in the ERAD. In this respect, it also remains unknown whether the OTU1 population involved in the ERAD directly associates with the ER.

## Methods

All methods can be found in the accompanying Transparent Methods supplemental file.

## Supplemental Information

Document S1. Transparent Methods, Figure S1, and Table S1
