# Supplementary material for: Histone Deubiquitinase OTU1 Epigenetically Regulates DA1 and DA2, Which Control Arabidopsis Seed and Organ Size
Source: iScience. 2020 Feb 28;23(3):100948. doi: 10.1016/j.isci.2020.100948 (PMC7068640; doi:10.1016/j.isci.2020.100948)
Supplement: Document S1. Transparent Methods, Figure S1, and Table S1 [file mmc1.pdf]

iScience, Volume *23*

Supplemental Information

Histone Deubiquitinase OTU1 Epigenetically Regulates *DA1* and *DA2*, Which Control *Arabidopsis* Seed and Organ Size

Ido Keren, Benoît Lacroix, Abraham Kohrman, and Vitaly Citovsky

SUPPLEMENTARY INFORMATION

TRANSPARENT METHODS

Plants

Seeds of the wild-type *Arabidopsis thaliana* (ecotype Col-0) plants and of the SALK_010551 and SALK_058652 lines, representing the *otu1-1* and *otu1-2* T-DNA insertional mutants, respectively, were obtained from the Arabidopsis Biological Resource Center (abrc.osu.edu). Seeds were surface-sterilized with 0.6% sodium hypochlorite and 70% ethanol, plated on MS medium (Murashige and Skoog, 1962) with 0.8% (w/v) agar, containing 3% (w/v) sucrose, stratified for 3 days at 4°C in the dark, transferred to a controlled environment growth chamber, grown at 22°C under long-day conditions (16-h light/8-h dark cycle at 100 $\mu E\ sec^{-1}m^{-2}$ light intensity), transferred to soil, and maintained under the same growth conditions.

Seed Weight, Size and Germination Time Course

For measuring weight, seeds were harvested from mature plants, dried at 24°C for 7 days and weighed. Ten batches of 50 seeds (N=500) from each line was weighed using Orion Cahn C-33 Microbalance (Thermo Scientific Inc.). For measuring surface area, dried seeds were recorded using a Leica MZ FLIII stereoscope and the area of each photographed seed was determined by ImageJ software (Fiji Life-Line version, 2014). For quantification of germination, the stratified seeds were grown at 22°C under long-day conditions, and the number of germinated seeds was recorded after 12 h, 24 h, 36 h, 2 days, 3 days, 4.5 days, and 6.5 days. The germination rate was expressed as percent of germinated seeds out of 500 total planted seeds (N=500).

Plant Organ Measurements

A metric ruler was used for all plant organ measurements. For stem length, 35 plant stems from each line were measured from the rosette area to the end of the stem at 35 days after

seed stratification (DAS) (N=35). For cotyledon length, 10 cotyledons from each line were measured at 7 DAS (N=10). For leaf rosette diameter, the distance between the ends of the two oldest leaves was measured in 50 plant leaf rosettes from each line at 21 DAS (N=50).

For imaging individual rosette leaves, a Sony A6000 camera equipped with a zoom Sony Kit lens (SELP 16 mm-50 mm f3.5-6.3) used to capture images. Each of the seven sequential leaves from a rosette of each line was removed and placed right-to-left sequentially for size comparison. Images are representative of multiple independent experiments (N=10 images from 3 plants of each line).

Microbombardment and Subcellular Localization

OTU1 was fused to CFP by inserting its coding sequence into the BglII/BamHI sites of pSAT6-ECFP-C1 (GenBank accession number AY818374) (Tzfira et al., 2005). Free mRFP was expressed from pSAT6-mRFP-C1 (Stock number CD3-1107, The Arabidopsis Information Resource, TAIR). The construct expressing Agrobacterium VirD2 NLS fused to mRFP has been described (Citovsky et al., 2006). Tested constructs were mixed in a molar ratio of 1:1, adsorbed onto 10 mg of 1-μm gold particles (Bio-Rad, CA) and bombarded at 100-120 psi into the leaf epidermis of greenhouse-grown *A. thaliana* using a Helios gene gun (PDS-1000/He, Bio-Rad) (Ueki et al., 2009). After incubation for 24-48 h at 22-24°C, the bombarded tissues were viewed under a Zeiss LSM 5 Pascal confocal laser scanning microscope.

Light Microscopy and Cell Size Measurements

Differential interference contrast (DIC) images were acquired using a CCD digital camera (Axiocam MRm, Carl Zeiss, Oberkochen, Germany) mounted on a microscope (Axioimager, Carl Zeiss) with a Plan-NeoFluar 20X/0.8 differential interference contrast objective controlled by Zen 2012 (Carl Zeiss) (Keren and Citovsky, 2016). The acquired images

were analyzed using ImageJ (Fiji Life-Line version, 2014) and Paint.NET software (version 4.0.6, dotPDN LLC). Cell size and surface density were measured in six different images, recorded with identical magnification, of the middle region of the fifth leaf blade at ca. 1.0 mm from the middle vein. Each measurement was performed in three biological replicates, each consisting of three technical replicates.

Quantitative RT-PCR (RT-qPCR)

For RT-qPCR analyses (Keren and Citovsky, 2016, 2017), total RNA was extracted either from aerial parts of 21-days-old plants or from the indicated plant tissues at the indicated ages using NucleoSpin RNA plant kit (MACHEREY-NAGEL GmbH & Co.). This RNA preparation (1 µg) was reverse transcribed, and the resulting cDNA preparation (2 µl for each sample) was amplified using Power SYBR Green PCR Master Mix (Thermo Fisher Scientific Inc.) and specific primers described in Table S1 in a StepOnePlus real-time PCR system (Applied Biosystems) for 1 cycle at 95°C for 5 min and 40 cycles each at 95°C for 10 s, 57°C for 10 s, and 72°C for 15 s. Unless indicated otherwise, each sample was analyzed in three biological replicates, each consisting of three technical replicates, using validated constitutive reference gene *UBQ10* (At4g05320) (Keren and Citovsky, 2016, 2017) to normalize RT-qPCR data by the comparative C_t method, with ΔC_t calculated by subtracting the C_t value of the tested transcript from the C_t value of *UBQ10* transcripts in each sample; the relative transcript levels were calculated by the cycle threshold (CT) $2^{-\Delta\Delta C_t}$ method (Livak and Schmittgen, 2001).

Quantitative Chromatin Immunoprecipitation (qChIP)

For ChIP analyses (Keren and Citovsky, 2016, 2017), cell nuclei were isolated from areal parts (~3 g) of 21-days-old plants, cross-linked by 1% formaldehyde (v/v), and sonicated to achieve chromatin shearing to an average size of 0.4-1.0 kb fragments. The resulting

preparations were incubated at 4°C for 1 h with protein A agarose beads (40 µl; 16-157, Millipore), centrifuged, and the supernatant was incubated at 4°C for overnight with the appropriate antibody [anti-acetyl-histone H3 (06-599, Millipore), anti-monoubiquityl-histone H2B (Lys-120) (5546S, Cell Signaling Technology, Inc.), or anti-trimethyl H3K4 (8580, Abcam)], combined with protein A agarose beads (60 µl), followed by additional 2-h incubation at 4°C. Then, the beads were washed sequentially with low and high salt buffers [20 mM Tris-HCl pH 8.0, 2 mM EDTA, 0.1% SDS, 1.0% Triton X-100 supplemented with 0.15 M NaCl (low salt) or 0.5 M NaCl (high salt)], LiCl buffer (250 mM LiCl, 10 mM Tris-HCl pH 8.0, 1.0 mM EDTA, 1% NP-40, 1.0% deoxycholate), and twice with TE (10 mM Tris-HCl pH 8.0, 1.0 mM EDTA), and eluted at room temperature for 15 min in the elution buffer (0.1 M $NaHCO_3$, 0.5% SDS). The cross-linking was reversed by incubation in 0.2 M NaCl at 65°C for overnight followed by digestion for 90 min at 45°C with Proteinase K (20 mg/ml). The recovered DNA (10 ng) was analyzed by qPCR using the appropriate primers (Table S1) as described above. The absence of non-specific, background signal was verified using protein A agarose incubated with chromatin samples in the absence of antibody.

Statistical Analyses

For RT-qPCR and qChIP experiments and for comparisons of seed weight and surface area, i.e., when the values in the wild-type plants are set to 1.0 or 100%, respectively, the corresponding quantitative data were analyzed by Wilcoxon signed-rank tests using Minitab 19 and the online tool at https://ccb-compute2.cs.uni-saarland.de/wtest/ (Marx et al., 2016). For organ/cell size measurements, the quantitative data were analyzed by Wilcoxon rank-sum tests. For seed germination, the quantitative data at the linear portion of the *otu1-1* and *otu1-2* gemination time course were analyzed by a Fisher’s exact test. p-values = 0.05, corresponding to

the statistical probability of 95%, were considered statistically significant. Standard deviation (SD) calculations were performed using Excel 2016 (Microsoft Inc.).

SUPPLEMENTAL REFERENCES

Citovsky, V., Lee, L.Y., Vyas, S., Glick, E., Chen, M.H., Vainstein, A., Gafni, Y., Gelvin, S.B., and Tzfira, T. (2006). Subcellular localization of interacting proteins by bimolecular fluorescence complementation *in planta*. J Mol Biol *362*, 1120–1131.

Keren, I., and Citovsky, V. (2016). The histone deubiquitinase OTLD1 targets euchromatin to regulate plant growth. Sci Signal *9*, ra125.

Keren, I., and Citovsky, V. (2017). Activation of gene expression by histone deubiquitinase OTLD1. Epigenetics *12*, 584-590.

Kumar, S., Stecher, G., and Tamura, K. (2016). MEGA7: Molecular Evolutionary Genetics Analysis version 7.0 for bigger datasets. Mol Biol Evol *33*, 1870-1874.

Livak, K.J., and Schmittgen, T.D. (2001). Analysis of relative gene expression data using real-time quantitative PCR and the 2(-delta delta C(T)) method. Methods *25*, 402-408.

Marx, A., Backes, C., Meese, E., Lenhof, H.-P., and Keller, A. (2016). EDISON-WMW: Exact Dynamic Programing Solution of the Wilcoxon-Mann-Whitney Test. Genomics Proteomics Bioinformatics *14*, 55-61.

Murashige, T., and Skoog, F. (1962). A revised medium for rapid growth and bio assays with tobacco tissue cultures. Physiol Plant *15*, 473-497.

Saitou, N., and Nei, M. (1987). The neighbor-joining method: a new method for reconstructing phylogenetic trees. Mol Biol Evol *4*, 406-425.

Tzfira, T., Tian, G.W., Lacroix, B., Vyas, S., Li, J., Leitner-Dagan, Y., Krichevsky, A., Taylor, T., Vainstein, A., and Citovsky, V. (2005). pSAT vectors: a modular series of plasmids for fluorescent protein tagging and expression of multiple genes in plants. Plant Mol Biol *57*, 503-516.

Ueki, S., Lacroix, B., Krichevsky, A., Lazarowitz, S.G., and Citovsky, V. (2009). Functional transient genetic transformation of *Arabidopsis* leaves by biolistic bombardment. Nat Protoc *4*, 71-77.

Zuckerkandl, E., and Pauling, L. (1965). Evolutionary divergence and convergence in proteins. In Evolving Genes and Proteins V. Bryson, and H.J. Vogel, eds. (Academic Press, New York), pp. 97-166.

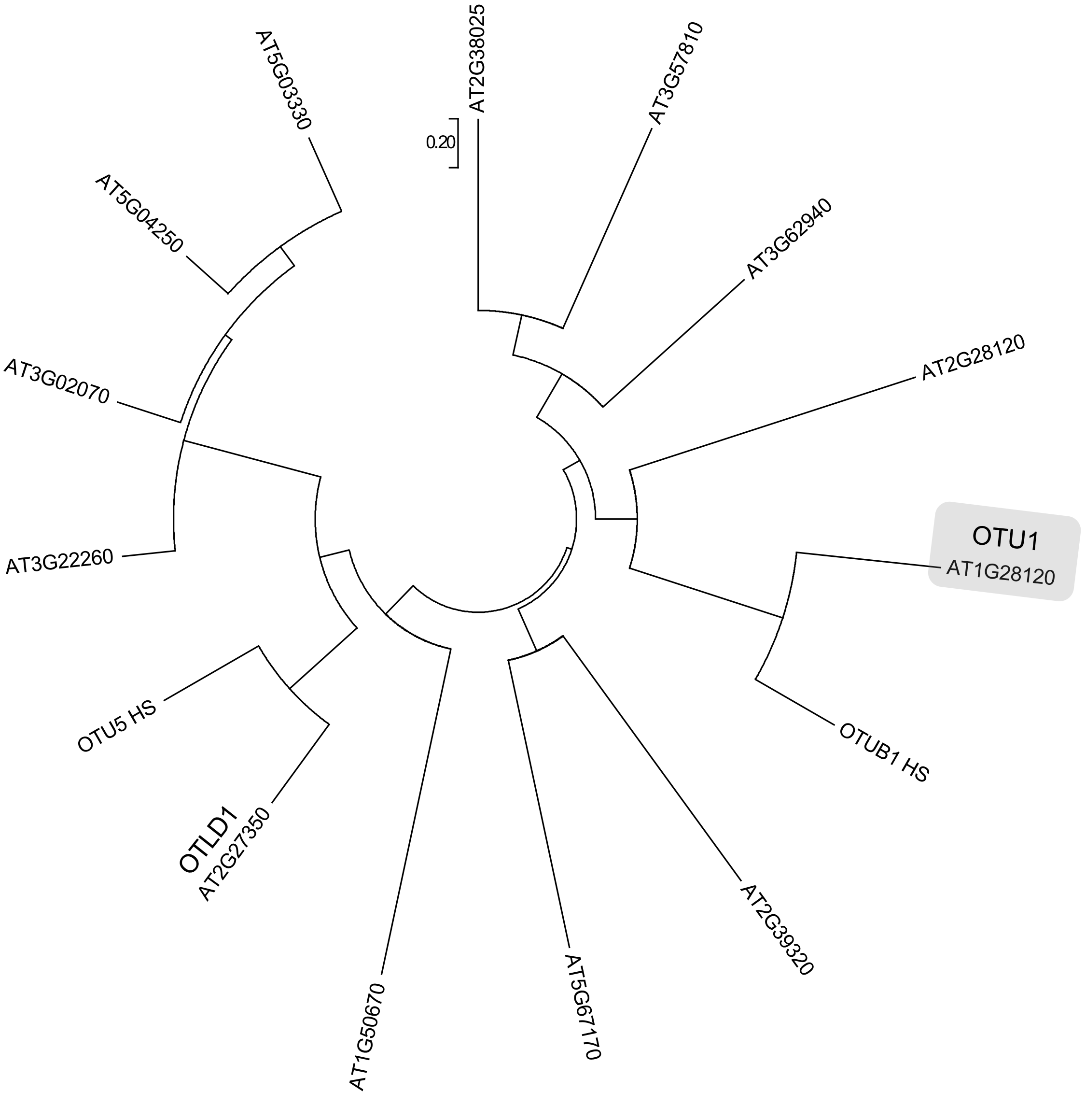
AT2G38025
0.20
AT3G57810
AT5G03330
AT5G04250
AT3G62940
AT2G28120
AT3G02070
OTU1
AT1G28120
AT3G22260
OTU5 HS
OTUB1 HS
OTLD1
AT2G27350
AT2G39320
AT1G50670
AT5G67170

Table S1. List of primers used in this study

Gene name and description	Primer name [a]	AGI locus identifier	Primer sequence (5' to 3')	Application
OTU1 - ubiquitin thioesterase otubain-like protein	SALK_010551-F	At1g28120 mutant (*otu1-1*)	AAGGTTACATTTAAAATGTACTTCCC	PCR
	SALK_010551-R		ACAATTTCCCCATTCTTCACC	
	SALK_058652-F	At1g28120 mutant (*otu1-2*)	TGCTTAGTGTTGGTTCCCAAG	PCR
	SALK_058652-R		AAAAAGGTGGTCGATTTACCG	
	AtOTU1-F	At1g28120	TTTGCAAGTCCTCGGTCGAA	RT-qPCR
	AtOTU1-R		ATTGCAACACCAAGTGCGTC	
SALK T-DNA - left border of the T-DNA insertion	SALK_LBb1.3	pROK2 T-DNA	ATTTTGCCGATTTCGGAAC	PCR
DA1 - ubiquitin-activated peptidase	DA1-F	At1g19270	GACACCATGCAATGCCAACC	RT-qPCR
	DA1-R		CTTTGAGCCTCATCCACGCA	
DA2 - RING-type E3 ubiquitin ligase	DA2-F	At1g78420	CATCATCATCGTCATCAT	RT-qPCR
	DA2-R		CATCATCATCTGTTCCTC	
BIG BROTHER (BB) - E3 ubiquitin ligase	BB-F	At3g63530	GGTGTGTGATATGCCAGCTC	RT-qPCR
	BB-R		CCATTTGGAAATGCATTCAG	
KLU - cytochrome P450 CYP78A5 monooxygenase	KLU-F	At1g13710	TGATTCTGACATGATTGCTGTTCT	RT-qPCR
	KLU-R		TCGCAACTGTATCTGTCCCTCTA	
GA20OX2 - gibberellin 20 oxidase 2	GA20ox2-F	At5g51810	ATGGCGTTTTTCTTGTGTCC	RT-qPCR
	GA20ox2-R		CCAATTCGAAAAGGAATCGA	
SHB1 - short hypocotyl under blue 1	SHB1-R	At4g25350	CATCCAAGCTTCCCGGAATAGGTCA	RT-qPCR
	SHB1-F		CCGCCGTCTCGAGCCCTTCT	
UBQ10 - ubiquitin 10	UBQ10-F	At4g05320	CGGAAAGCAGTTGGAGGATGG	RT-qPCR
	UBQ10-R		CGGAGCCTGAGAACAAGATGAAG	
DA1 - ubiquitin-activated peptidase	DA1-A-F	upstream of At1g19270	AGGCTGCATTGCCGTATGA	qChIP
	DA1-A-R		TATTCCCAACCCGGAGCCTT	
	DA1-B-F		TCCGTTTGGAACTCGTTTGCT	
	DA1-B-R		CAGCCTGCAAAATCGTCGAA	
	DA1-D-F		AGCACATTCTGGGTTTATTCGT	
	DA1-D-R		TCAAGCAAGGGAAGCAGCAA	
DA2 - RING-type E3 ubiquitin ligase	DA2-A-F	upstream of At1g78420	CGCAGGTTATGTGGTGGAGG	qChIP
	DA2-A-R		ACCACTTGCCTCTTCCTTCC	
	DA2-B-F		TGTAACCAGCCCCGAATTGA	
	DA2-B-R		ACCTCCACCACATAACCTGC	

	DA2-C-F		CGTCTCTTGTTTTCTTCTGCCC	
	DA2-C-R		ACACAATTGGGGCAAAACCC	
	DA2-G-F		GCGTAAATGGCTGAGGCAAA	
	DA2-G-R		CGTGAGTGTGTTTTGGGTTGA	
BIG BROTHER (BB) - E3 ubiquitin ligase	BB-B-F	upstream of At3g63530	ACTTTCCCGGCATCCATACG	qChIP
	BB-B-R		TGGAGAAACTCTTGGGCGTG	

(a) F, forward primer; R, reverse primer; A-C and F-G, the corresponding promoter regions tested in qChIP analyses.

SUPPLEMENTARY FIGURE AND TABLE

Fig. S1. Phylogenetic Tree of the Arabidopsis OTU Family of Deubiquitinases, Related to Figure 1. For comparison, the type-member of the human OTU enzymes, otubain-1 (OTUB1) and another human OTU enzyme, OTU5, were used. OTU1 (At1g28120) is highlighted by a shaded box. The evolutionary history was inferred using the Neighbor-Joining method (Saitou and Nei, 1987). The optimal tree with the sum of branch length = 13.22000264 is shown. The tree is drawn to scale, with branch lengths in the same units as those of the evolutionary distances used to infer the phylogenetic tree. The evolutionary distances were computed using the Poisson correction method (Zuckerkandl and Pauling, 1965) and are in the units of the number of amino acid substitutions per site. The analysis involved 15 amino acid sequences. All positions containing gaps and missing data were eliminated. There were a total of 161 positions in the final dataset. Evolutionary analyses were conducted in MEGA7 in Molecular Evolutionary Genetics Analysis tool (MEGA, version 7 for Mac OS X) (http://www.megasoftware.net) (Kumar et al., 2016). Scale bar, 0.20 amino acid substitutions per site.

Table S1. List of Tested Genes and Corresponding PCR Primers, Related to Figures 1 and 6-9 and Transparent Methods.
